# Association between preoperative serum albumin and long-term all-cause mortality after percutaneous vertebroplasty for vertebral compression fracture

**DOI:** 10.1371/journal.pone.0352159

**Published:** 2026-06-24

**Authors:** Yu-Tsung Lin, Wen-Chien Wang, Yu-Hsien Lin, Yun-Che Wu, Kun-Hui Chen, Chien-Chou Pan, Ching-Heng Lin, Jun-Sing Wang, Cheng-Hung Lee

**Affiliations:** 1 Taichung Veterans General Hospital, Taichung, Taiwan; 2 Department of Post-Baccalaureate Medicine, College of Medicine, National Chung Hsing University, Taichung, Taiwan; 3 Department of Computer Science and Information Engineering, Providence University, Taichung, Taiwan; 4 Department of Rehabilitation Science, Jenteh Junior College of Medicine, Nursing and Management, Miaoli, Taiwan; 5 Department of Medical Research, Taichung Veterans General Hospital, Taichung, Taiwan; 6 Division of Endocrinology and Metabolism, Department of Internal Medicine, Taichung Veterans General Hospital, Taichung, Taiwan; 7 Department of Food Science and Technology, Hung Kuang University, Taichung, Taiwan; National Trauma Research Institute, AUSTRALIA

## Abstract

**Purpose:**

Albumin levels have demonstrated superior predictive value for surgical outcomes across a variety of surgeries. However, their effect on long-term survival after vertebroplasty for vertebral compression fracture has not yet been well studied. This study aims to investigate the relationship between preoperative albumin levels and long-term survival in patients who underwent percutaneous vertebroplasty for vertebral compression fractures.

**Materials and methods:**

We enrolled patients who underwent vertebroplasty for vertebral compression fracture between the period of May 1, 2013 to June 30, 2020 in a single medical center. Patients who were diagnosed with a pathologic compression fracture, underwent additional spinal instrumentation during the same surgery, and those without data regarding their bone mineral density (BMD) were excluded. Cox proportional hazard models were conducted to examine the effects of hypoalbuminemia (<3.5 g/dL) on all-cause mortality after adjusting for age, gender, body mass index, smoking, diabetes, hypertension, chronic kidney disease (CKD), osteoporosis.

**Results:**

A total of 145 patients were analyzed, and the median follow-up period was 2.12 (interquartile range 1.51–3.28) years (mean follow-up 2.65 [SD 1.78] years). Compared with normal albumin levels, patients with hypoalbuminemia were found to be independently associated with a higher risk of all-cause mortality (hazard ratio 3.154, p = 0.014). These findings remained significant when albumin was examined as a continuous variable (hazard ratio 0.242, p = 0.001) and even after multivariate adjustment as well (hazard ratio 0.192, p = 0.004).

**Conclusion:**

Hypoalbuminemia was associated with all-cause mortality among patients who underwent vertebroplasty for vertebral compression fractures. Our findings highlighted the impact of nutritional status on long-term mortality in an elderly surgical population.

## Introduction

Vertebral compression fracture is one of the most common fragility fractures, leading to impaired functional status and increased mortality rates [1,2]. Individuals with one or more vertebral body fractures experience notably higher mortality risks when compared to those without fractures, determined as a 4.4 times greater risk. Consequently, it becomes crucial to identify predictive factors for adverse outcomes in this patient population [3]. While nonoperative management through the use of analgesic agents together with back brace therapy is a viable approach for vertebral compression fracture [4], a considerable proportion of patients fail to achieve adequate pain relief through conservative treatment [5]. Percutaneous vertebroplasty therefore emerges as a safe surgical procedure involving both a short surgical time and hospital stay, while also providing immediate pain relief and a rapid return of functional independence [6,7]. Each year, approximately 80,000 cases of vertebral augmentation are performed in the United States [8]. Despite the large number of surgeries conducted in recent decades, factors associated with long-term survival after vertebroplasty remain unknown.

Albumin levels have been proven to be a superior predictor of surgical outcomes when compared to many other preoperative patient characteristics across a variety of surgeries [9–11]. Considering the pivotal role which nutritional status plays in the fragile elderly population, where it reflects on ones overall health condition, albumin levels have become a routine screening tool for nutritional assessment [12]. This study aims to investigate the relationship between preoperative albumin levels and long-term survival in patients who underwent percutaneous vertebroplasty for vertebral compression fractures.

## Materials and methods

This study was an analysis of retrospectively collected data in a single medical center. We enrolled patients who underwent percutaneous vertebroplasty for single-level thoracolumbar compression fracture in the Department of Orthopedics at our hospital between May 1, 2013 and June 30, 2020. The survival status of patients was confirmed on March 30, 2021, using de-identified and anonymized data obtained from the Ministry of Health and Welfare, R.O.C. (Taiwan). This study was conducted in accordance with the Declaration of Helsinki and approved by the Institutional Review Board of our hospital (Approval number: CE22167A). The IRB also approved the waiver of informed consent due to the retrospective nature of the study using extracted medical records data.

Demographic data was collected, including age, gender, body mass index, smoking status, and operation level. Laboratory data for preoperative assessment, including serum albumin level, and patients’ medical comorbidity (including diabetes mellitus, hypertension, and chronic kidney disease) were obtained from the available electronic medical records. Chronic kidney disease was defined as having an estimated glomerular filtration rate <60 mL/min/1.73 m² at baseline. The diagnosis of osteoporosis, defined as having a T-score ≤ −2.5 according to Dual-energy X-ray absorptiometry or the use of medications for osteoporosis (including bisphosphonate, receptor activator of nuclear factor kappa-B inhibitor, or parathyroid hormone) was reviewed.

**Fig 1:**
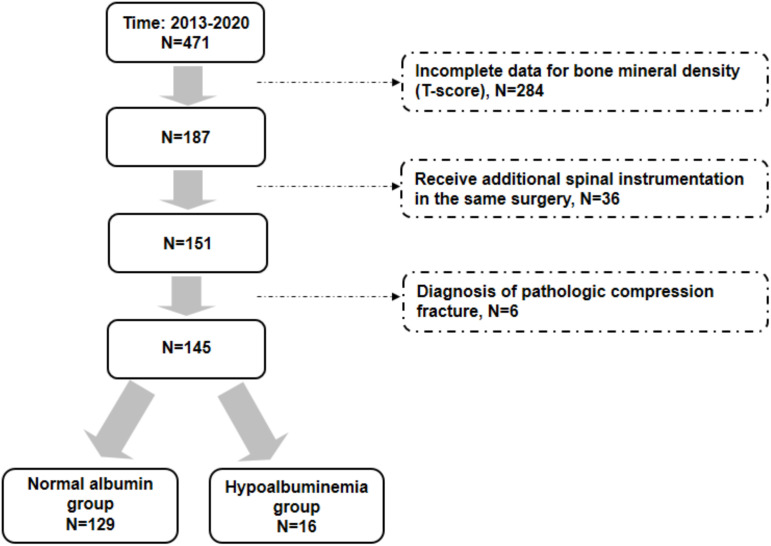
Flow chart for patient enrollment.

Fig 1 shows the inclusion of study patients. We retrospectively collected data on 471 patients who underwent percutaneous vertebroplasty for a single-level thoracolumbar compression fracture between the years 2013 and 2020. We excluded patients who had no available data regarding their bone mineral density (n = 284), had additional spinal instrumentation during the same surgery (n = 36), and who had a diagnosis of pathologic compression fracture (n = 6). A total of 145 patients were eventually included in the analysis. We defined hypoalbuminemia as a serum albumin level < 3.5 g/dL [13]. All study patients were divided into two groups according to their preoperative serum albumin level (< 3.5 g/dL vs. ≥ 3.5 g/dL).

### Statistical analysis

The statistical differences in baseline variables between the two groups were assessed using the student t-test for continuous variables, while the chi-square test was used for categorical variables. We calculated the number of patients required to provide a statistical power of 90% to detect a fourfold higher mortality risk associated with hypoalbuminemia [14] with a 2-sided significance level of 0.05. The 1-year mortality rate in patients who underwent vertebroplasty for vertebral compression fracture in previous studies [15,16] was around 5%. We estimated that patients with hypoalbuminemia may have a 1-year morality rate of 20%. This calculation led to a sample size of 39. Cumulative survival curves of the two groups were examined using the Kaplan–Meier method. Cox regression analysis was conducted to examine the effect of hypoalbuminemia (albumin < 3.5 g/dL) and serum albumin level (as a continuous variable) on all-cause mortality with adjustments for age, gender, body mass index, smoking, diabetes, hypertension, chronic kidney disease, osteoporosis, and medical treatment osteoporosis. The Scaled Schoenfeld residuals were used to test the assumption of Cox-proportional hazards model, and no violation of the assumption was confirmed. A restricted cubic spline plot was used as a sensitivity test to explore the relationship between serum albumin concentrations (as a continuous variable) and all-cause mortality. All statistical analyses were performed using the Statistical Package for the Social Sciences (IBM SPSS Statistics for Windows, version 22.0; International Business Machines Corp., NY, USA). The level of statistical significance was set at p < 0.05.

## Results

Of the 145 patients included in the analysis (mean age 76.2 ± 9.2 years, male 27.6%, mean BMI 23.9 ± 4.1 kg/m^2^, Table 1), 16 had a preoperative serum albumin level < 3.5 g/dL. The mean serum albumin level was 3.1 ± 0.3 g/dL in these patients. In contrast, patients with a preoperative serum albumin level ≥ 3.5 g/dL had a mean serum albumin level of 4.0 ± 0.3 g/dL. The former group had a higher rate of chronic kidney disease (50.0% vs. 34.1%, p = 0.007), and a lower rate of medication use for osteoporosis than the latter (37.5% vs. 64.3%, p = 0.038). There were no significant between-group differences seen in the other variables (Table 1).

**Table 1:** Baseline characteristics of the study population according to serum albumin.

Variables	< 3.5 g/dL	≥ 3.5 g/dL	P value
N	16	129	
Age, years	79.5 ± 8.0	75.8 ± 9.3	0.133
Male gender, n (%)	6 (37.5)	34 (26.4)	0.347
Body mass index, kg/m^2^	24.2 ± 4.8	23.9 ± 4.1	0.788
Smoking, n (%)	2 (12.5)	9 (7.0)	0.431
Diabetes, n (%)	5 (31.3)	25 (19.4)	0.269
Hypertension, n (%)	9 (56.3)	65 (50.4)	0.658
CKD, n (%)^a^	8 (50.0)	44 (34.1)	0.007
CKD stage 3	6 (37.5)	35 (27.1)	
CKD stage 4	0	5 (3.9)	
CKD stage 5	2 (12.5)	4 (3.1)	
Serum albumin, g/dL	3.1 ± 0.3	4.0 ± 0.3	<0.001
Osteoporosis, n (%)	12 (75.0)	95 (73.6)	0.907
Medication for osteoporosis, n (%)^b^	6 (37.5)	83 (64.3)	0.038
Level of vertebroplasty, n (%)			0.932
T-spine	7 (43.8)	55 (42.6)	
L-spine	9 (56.3)	74 (57.4)	

Values are mean ± SD or n (%). CKD, chronic kidney disease. CKD stage 3, estimated glomerular filtration rate 30 to <60 mL/min/1.73 m². CKD stage 4, estimated glomerular filtration rate 15 to <30 mL/min/1.73 m². CKD stage 5, estimated glomerular filtration rate <15 mL/min/1.73 m². ^a^estimated glomerular filtration rate <60 mL/min/1.73 m². ^b^Bisphosphonate, receptor activator of nuclear factor kappa-B inhibitor, or parathyroid hormone.

**Fig 2:**
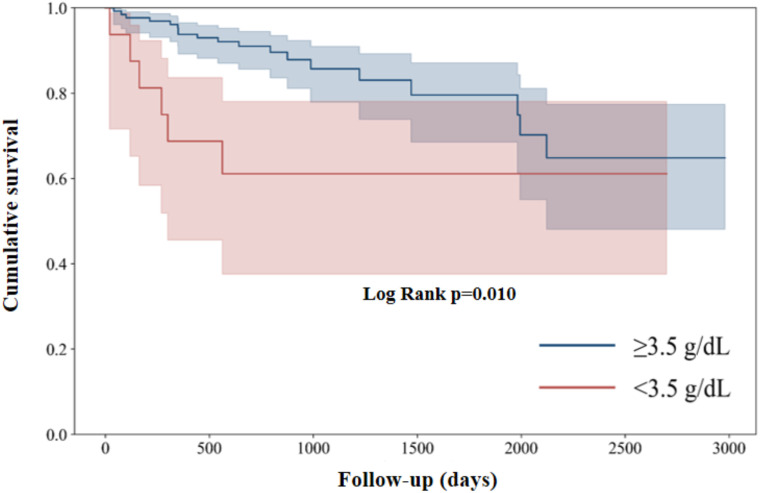
Kaplan–Meier survival curves with 95% confidence intervals (shaded zones). Survival curve was stratified by serum albumin level. Patients with albumin levels <3.5 g/dL had significantly lower survival compared to those with levels ≥3.5 g/dL (log-rank test, p = 0.010).

Fig 2 shows the Kaplan-Meier survival curves of the two groups. A total of 25 patients died during the median follow-up period of 2.12 (interquartile range 1.51–3.28) years (mean follow-up 2.65 [SD 1.78] years). Patients with a preoperative albumin level < 3.5 g/dL had a lower survival rate than those who had a preoperative albumin level ≥ 3.5 g/dL (Log rank p = 0.010). Analysis of cox proportional hazard model revealed the higher risk of all-cause mortality in the hypoalbuminemia group (hazard ratio 3.154, 95% CI 1.256 to 7.979, p = 0.014, Table 2). The finding remained significant after multivariate adjustment (hazard ratio 3.569, 95% CI 1.275 to 9.988, p = 0.015).

**Table 2:** Association of pre-operative serum albumin with all-cause mortality.

	Hazard Ratio (95% CI)	P
Serum albumin (< 3.5 g/dL vs. ≥ 3.5 g/dL)		
Model 1	3.154 (1.256-7.919)	0.014
Model 2	2.902 (1.136-7.410)	0.026
Model 3	3.569 (1.275-9.988)	0.015
Serum albumin (g/dL)		
Model 1	0.242 (0.103, 0.570)	0.001
Model 2	0.250 (0.097, 0.639)	0.004
Model 3	0.192 (0.062, 0.598)	0.004

Model 1, unadjusted. Model 2, adjusted for age and gender. Model 3, adjusted for variables in Model 2 plus body mass index, smoking, diabetes, hypertension, chronic kidney disease, osteoporosis, and medical treatment for osteoporosis.

**Fig 3:**
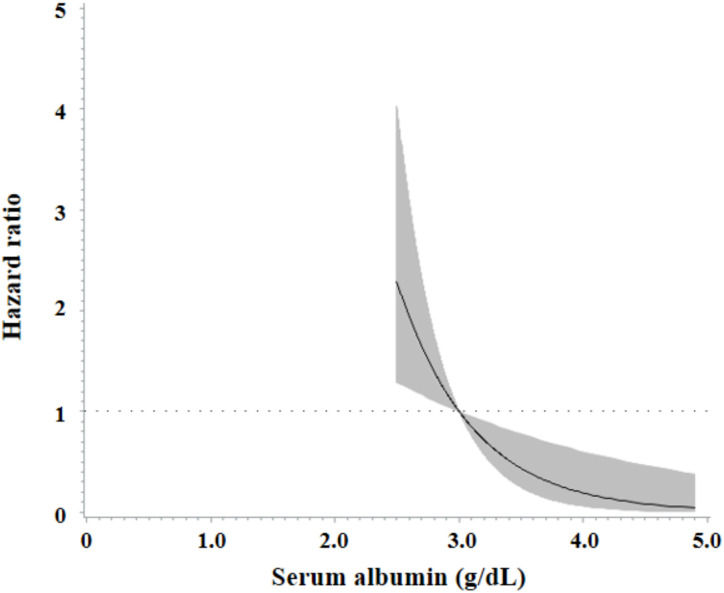
Cubic spline analysis. Sensitivity analysis using cubic spline of preoperative albumin levels versus the risk of postoperative long-term all-cause mortality. Significant increase in all-cause mortality was found when albumin level was below 3.0 g/dL.

The findings were consistent when albumin was treated as a continuous variable. The analysis revealed that the level of preoperative serum albumin was associated with a lower risk of all-cause mortality (hazard ratio 0.242, 95% CI 0.103 to 0.570, p = 0.001), the finding remained significant even after multivariate adjustment (hazard ratio 0.192, 95% CI 0.062 to 0.598, p = 0.004, Table 2). Fig 3 shows the cubic spline of serum albumin versus risk of mortality. The level of serum albumin below which there was a significant increase in all-cause mortality was around 3.0 g/dL.

## Discussion

Malnutrition has been recognized as a predictor of adverse outcomes in both elective and nonelective orthopedic surgeries [17–21]. However, the relationship between nutritional status and long-term mortality following percutaneous vertebroplasty for vertebral compression fracture has received limited attention in previous literature. In the present study, we demonstrated that hypoalbuminemia (preoperative albumin < 3.5 g/dL) was associated with an increase in all-cause mortality after a median follow-up duration of more than 2 years. It is noteworthy that a significant rise in mortality risk was observed among patients with a preoperative albumin level < 3.0 g/dL (Fig 3).

Hypoalbuminemia has been identified as a significant factor associated with increased postoperative complications and mortality in patients suffering from fragility fractures [22,23]. In a large retrospective cohort study involving 29,377 patients, Daniel et al. [24] demonstrated that hypoalbuminemia is an independent risk factor for mortality following surgery for geriatric hip fractures. This highlights the critical role of nutritional status, as indicated by albumin levels, in determining the outcomes of surgical interventions in this vulnerable patient population. This association extends beyond hip fractures, as hypoalbuminemia was also found to be associated with higher postoperative complications and mortality in other fragility fractures, including distal radius fractures and proximal humeral fractures [25,26]. Our findings further support this association, as we found that an albumin level below 3.5 g/dL was associated with increased all-cause mortality in patients who underwent vertebroplasty for vertebral compression fracture. The aforementioned results are consistent with a recent systematic review and meta-analysis [27] which revealed that baseline hypoalbuminemia (<3.5 g/L) is significantly associated with postoperative readmission, reoperation, and mortality among orthopedic trauma patients. Moreover, Kim HJ et al. [14] reported that albumin level <3.0 g/dL was associated with major complications and mortality after vertebroplasty or kyphoplasty for osteoporotic vertebral compression fracture.

Being one of the most prevalent fragility fractures, vertebral compression fracture is known to carry a significant risk of mortality. Studies have indicated high mortality rates of up to 59.9% at 3 years and 30.9% at 5 years [28]. Vertebral compression fractures commonly occur in the elderly after a simple fall from standing height, which typically does not result in serious systemic injury. Although a compression fracture itself is generally considered non-fatal, it can lead to a detrimental cascade of symptoms and morbidity, ranging from pain and disability to impaired pulmonary and respiratory function [3]. Therefore, the observed increased mortality associated with vertebral compression fracture in elder populations may be partly attributed to each patient’s comorbidities [29]. Our findings have revealed a notable association between mortality and low albumin levels. This highlights the substantial impact which severe malnourishment has on the elderly with regards to postoperative long-term mortality.

The surgical treatment of vertebral compression fracture using percutaneous vertebroplasty has been a controversial subject over the past decades, particularly after a multicenter randomized control trial revealed that there were no clear benefits to the procedure in patients having compression fractures [30]. Nevertheless, a subsequent study based on a large Medicare dataset indicated that vertebroplasty could lead to a noteworthy 7% decrease in mortality rate when compared to non-surgical management in patients with vertebral compression fractures [31]. Furthermore, a meta-analysis involving more than 2 million patients demonstrated that those with osteoporotic vertebral compression fractures who underwent vertebral augmentation were 22% less likely to die at up to 10 years after treatment than those who received nonsurgical treatment [32]. These findings suggest that in fragile elderly populations, pain relief through the use of vertebroplasty may play a crucial role in preventing the disability and deterioration of one’s physical status, ultimately improving survival rates following the intervention. However, factors affecting survival after vertebroplasty have not been well studied. Our study demonstrated that in severely malnourished patients with a preoperative albumin level < 3.0 g/dL, the risk of mortality remained high despite the potential benefit of symptom relief after the procedure.

The significance of malnutrition on the treatment outcomes of vertebral compression fractures deserves further investigation. Ohba et al. [33] conducted a study investigating factors influencing mortality after surgical management, including posterior spinal instrumentation and balloon kyphoplasty, for insufficient union following vertebral compression fractures. They identified albumin levels below 3.5 g/dl as being the most significant risk factor associated with increased mortality during a mean follow-up period of 28.3 months. Similarly, Gupta et al. reported that preoperative serum albumin levels correlated with complications, re-admission rates and mortality following surgical intervention for osteoporotic vertebral compression fractures [34]. Consistent with these studies, our results revealed an increased long-term mortality risk in patients with hypoalbuminemia. To the best of our knowledge, this is the first study to investigate the association between hypoalbuminemia and long-term survival after percutaneous vertebroplasty for vertebral compression fractures.

Our study has several limitations. First, this was a retrospective, single-center study, and the sample size was relatively small. Hence, our study is hypothesis-generating, rather than confirmatory. Second, we do not have data on cause-specific mortality. Third, we did not collect data on chronic disease control, such as diabetes, hypertension, chronic kidney disease, albuminuria, inflammatory markers, and frailty assessment, all of which may have confounded all-cause mortality risk in our patients. Despite these limitations, our findings highlight the importance of nutritional status on long-term outcomes in patients who underwent percutaneous vertebroplasty for vertebral compression fracture. Addressing and managing malnutrition might improve postoperative recovery and long-term prognosis in these patient populations.

## Conclusion

We reported that pre-operative hypoalbuminemia (<3.5g/dL) was associated with all-cause mortality amongst patients who underwent vertebroplasty for vertebral compression fractures. Our findings highlight the impact of nutritional status on long-term mortality in elderly surgical populations. Larger, prospective, multicenter investigations are needed to validate the role of albumin as an independent predictor of postoperative mortality.
